# Factors associated with a healthy diet and willingness to change dietary behavior in older adults at increased risk of dementia

**DOI:** 10.1177/13872877251330296

**Published:** 2025-04-15

**Authors:** Iris Blotenberg, Andrea E Zülke, Melanie Luppa, Felix Wittmann, Thomas Fankhänel, Solveig Weise, Juliane Döhring, Catharina Escales, Robert P Kosilek, Irina Michel, Christian Brettschneider, Anke Oey, Birgitt Wiese, Jochen Gensichen, Hans-Helmut König, Thomas Frese, Hanna Kaduszkiewicz, Wolfgang Hoffmann, Steffi G Riedel-Heller, René Thyrian

**Affiliations:** 1 German Centre for Neurodegenerative Diseases (DZNE), Greifswald, Germany; 2 Institute of Social Medicine, Occupational Health and Public Health (ISAP), University of Leipzig, Leipzig, Germany; 3 Institute of General Practice and Family Medicine, Martin-Luther-University Halle-Wittenberg, Halle, Saale, Germany; 4 Institute of General Practice, University of Kiel, Kiel, Germany; 5 Institute of General Practice and Family Medicine, University Hospital, LMU Munich, Munich, Germany; 6 Department of Health Economics and Health Service Research, University Medical Centre Hamburg-Eppendorf, Hamburg, Germany; 7 State Health Department of Lower Saxony, Hannover, Germany; 8 MHH Information Technology – Science & Laboratory, Hannover Medical School, Hannover, Germany; 9 Institute for Community Medicine, University Medicine Greifswald, Greifswald, Germany; 10 Faculty V: School of Life Sciences, University of Siegen, Siegen, Germany

**Keywords:** aged, Alzheimer's disease, diet, healthy aging, motivation, primary prevention, self efficacy, transtheoretical model

## Abstract

**Background:**

Healthy dietary patterns have been linked to reduced risks for cardiovascular diseases and dementia, making nutrition an essential part of a comprehensive approach for dementia prevention. Knowledge about factors associated with a healthy diet in people with increased dementia risk is scarce.

**Objective:**

To analyze dietary habits and associated factors in older adults with increased dementia risk in Germany.

**Methods:**

We used baseline-data of the AgeWell.de-trial (n = 1001, %_female _= 52.2, M_age _= 69.0, SD = 4.9). Nutrition was assessed using a composite score, comprising 11 components covered by national recommendations for a healthy diet (range = 0–11 points). Linear regressions assessed associations of sociodemographic, social, health-related and psychological factors with consumption of a healthy diet. Further, we assessed stages of change based on the transtheoretical model of behavior change.

**Results:**

Consumption of a healthy diet was moderate (Median = 4, IQR = 2). Female sex (b = 0.64, 95% CI: 0.41, 0.88), higher levels of motivation for healthy eating (b = 0.22, 95% CI: 0.10, 0.34) and higher self-efficacy (b = 0.33, 95% CI: 0.20, 0.46) were linked to a healthy diet. Regarding the stages of behavior change, the majority were in the maintenance stage (45.2%), followed by the contemplation (21.5%) and precontemplation (21.2%) stages.

**Conclusions:**

Results suggest room for improvement regarding a healthy diet in our sample. Lifestyle-based interventions in older adults should be tailored towards current levels of motivation and self-efficacy of participants. Including modules targeting motivation and self-efficacy might help maximize intervention effectiveness.

## Introduction

The increasing number of people with dementia worldwide constitutes a major public health challenge.^1^ According to current estimates, around 1.8 million people in Germany were living with dementia in 2021,^2^ and due to demographic changes, these numbers are expected to continue to rise.^3^ In the absence of a causal therapy for Alzheimer's disease and related dementias, preventive efforts play a pivotal role in reducing incident cases and providing dementia risk reduction on a scalable level.^4,5^ Certain lifestyle factors are known to reduce the risk of dementia, including physical, social and cognitive activity^4^ as well as a healthy diet.^5–9^

Current evidence on the impact of nutrients, foods and dietary patterns on cognitive decline and dementia mostly stems from observational studies, with evidence from randomized controlled trials being less conclusive.^10–12^ Dietary patterns linked to lower dementia risk include the Mediterranean diet, or the Dietary Approaches to Stop Hypertension (DASH)-diet. Recent studies further reported links between plant-based dietary patterns and lower risk of Alzheimer's disease and other types of dementia.^13,14^ Effects of foods and nutrients on dementia might act in two ways, i.e., due to direct effects of specific foods and nutrients on the brain, and indirect effects on diseases such as diabetes or hypertension, which increase dementia risk.^6^ While definitions of “healthy diet” can vary across different cultures and local contexts, many foods, nutrients and dietary patterns are emphasized in several dietary approaches or nutritional guidelines. Current dietary recommendations for risk reduction of dementia and other diseases unequivocally emphasize daily intake of fruit and vegetables, low intake of salt and sugar, preferring wholegrain- over other grain products, and intake of plant-based fats, e.g., olive oil or nuts, and low to moderate alcohol consumption.^10,15,16^

Previous studies have identified some risk factors for unhealthy eating habits among older adults, among them sociodemographic factors like male sex, lower education and lower income.^17,18^ Low income can act as a barrier to the consumption of healthy, fresh foods due to their often higher cost.^18^ Being married has been linked to both healthier and unhealthier diets, as shown in two French studies in older adults.^19,20^ Social support has been linked to healthier diets, while mobility- and cognitive impairment tend to be associated with less healthy eating habits.^18^ Further strong predictors of healthy eating habits include motivation for healthy eating and self-efficacy.^21^

To increase the effectiveness of prevention measures, they should be tailored to an individual's risk profile.^22^ Identifying factors associated with healthy or unhealthy eating habits can help identify individuals who may benefit most from nutritional interventions and may help tailoring nutritional interventions to their needs. Of particular interest may be the group of older adults at increased risk of dementia, as this group may benefit especially from lifestyle interventions.^23^ However, factors associated with healthy eating in people with an increased risk of dementia have not yet been explored.

For the development and implementation of interventions to promote a healthy diet, it is important to gather knowledge about the target population's stage of behavior change. Behavior change is a complex process, which has been described in social cognitive models like the transtheoretical model of health behavior change (TTM;^24,25^). TTM conceptualizes behavior change as a sequence of five stages: People in the (1) precontemplation stage do not intend to change their behavior; those in the (2) contemplation stage intend to change their behavior, but are also acutely aware of the costs; those in the (3) preparation stage have already taken some action and intend to change their behavior in the near future; and finally, both people in the (4) action and (5) maintenance stages have already changed their behavior, but those in the maintenance stage have a lower temptation to relapse.^25^ In the TTM, Bandura's concept of self-efficacy plays a central role: the confidence in being able to perform a desired behavior is important for successfully navigating through the stages of change. Although interventions should be tailored to the target population's stage of behavior change, there is so far a lack of studies that investigate the stages of change in older adults with increased risk of dementia.

The *first objective* of the present study was to explore sociodemographic, social, health-related and psychological factors associated with a healthy diet in older adult at risk of developing dementia. Our *second objective* is to describe the stages of behavior change regarding healthy dietary habits in our sample of older adults at increased risk of dementia.

## Methods

### The AgeWell.de-trial and participants

This study uses data from the cluster-randomized AgeWell.de-trial, testing effectiveness of a multicomponent lifestyle-based intervention on cognitive performance and several secondary outcomes in a sample of older adults at increased risk for dementia according to a Cardiovascular Risk Factors, Aging and Incidence of Dementia (CAIDE;^26^)-risk score ≥ 9 points in Germany. Participants were recruited via their attending general practitioner (clusters). Baseline examinations took place between 06/2018 and 10/2019, follow-up assessments were conducted between 07/2020 and 01/2022. Study design and rationale,^27^ baseline characteristics of participants^28^ and trial results for primary and secondary outcomes^29^ have been described elsewhere. In short, the multicomponent intervention (optimization of nutrition and medication, enhancement of physical, social, and cognitive activity) did not improve global cognitive performance, while beneficial effects were detected for health-related quality of life in the total sample. In women, the intervention decreased depressive symptoms.^30^ AgeWell.de was prospectively registered in the German Clinical Trials Register (DRKS; trial identifier: DRKS00013555).

### Outcomes and covariates

During the baseline visit, participants filled out a validated food frequency questionnaire (FFQ;^31^), capturing frequency and amount of 53 food items consumed during the past four weeks. Frequency categories were: never; once a month; two to three times a month; one to two times a week; three to four times a week; five to six times a week; one time per day; two times per day; three times per day; four to five times per day, and more than five times per day. Building on an approach by Voortman and colleagues^32^ who calculated a sum score for healthy nutrition based on the Dutch dietary guidelines, we calculated a sum-score based on 11 components of a healthy diet (consumption of fruit and vegetables; whole-grain products; legumes; nuts; fish; tea; dairy products; red/processed meat; sugar-containing beverages; alcohol consumption). We applied scoring algorithms provided by the authors of the FFQ^31^ to account for differences in units of measurement for drinks (in ml) and foods (in g), allowing to calculate consumption within specific food categories (e.g., dairy products). For each item, a point was given if the respective item was fulfilled, leading to a total score with a possible range from 0 to 11. Operationalization of all items comprised by the healthy diet score is described in Table 1. To the best of our knowledge, no comparable scoring system based on the guidelines of the German Nutrition Society (DGE) is currently available. However, the Dutch and German guidelines for healthy nutrition are highly comparable in terms of content and recommendations.^16^ The Dutch dietary guidelines were designed based on systematic reviews and meta-analyses and aimed to provide recommendations that could contribute to lower risk of several chronic diseases, including heart disease, diabetes and dementia.^33^ While better adherence to the guidelines was not linked to lower risk for diabetes, heart disease and dementia in the population-based Rotterdam Study, associations have been shown with known risk factors for dementia, e.g., depression and stroke.^32^ Moreover, better adherence to the healthy diet score proposed by Voortman and colleagues was linked to larger brain volume, gray and white matter volume, and hippocampal volume.^34^

**Table 1.** Assessment of a healthy diet in the AgeWell.de-study.

Recommendation in the 2015 Dutch Dietary guidelines	Operationalization, based on Voortman et al. 2017	AgeWell.de – FFQ-item(s)	Points
Eat at least 200 g of vegetables daily	Vegetables ≥200 g/day	Total amount of vegetables, raw or cooked	1
Eat at least 200 g of fruit daily	Fruit ≥200 g/day	Total amount of fruit	1
Eat at least 90 g brown bread, whole-meal bread or other wholegrain products daily	Whole grain products ≥90 g/day	Whole-grain bread buns, slices of bread	1
Eat legumes weekly	Legumes ≥135 g/week	Legumes (including, e.g., lentils, chickpeas)	1
Eat at least 15 g of unsalted nuts daily	Nuts ≥15 g/day	Nuts	1
Eat one serving of fish, preferably oily fish, weekly	Fish ≥100 g/week	Fish, served cold; fish, served warm	1
Drink three cups of tea daily	Tea ≥ 450 ml/day	Black and green tea	1
Take a few portions of dairy products daily	Dairy ≥350 g/day	milk, cheese, cottage cheese/curd	1
Limit the consumption of red meat, particularly processed meat	Red and processed meat <300 g/week	Sausages, red meat, ham, meat-based fast food, e.g., hamburgers, kebab	1
Minimize the consumption of sugar-containing beverages	Sugar-containing beverages <150 ml/day	Sugar-containing beverages, e.g., lemonade	1
Do not drink alcohol or no more than one glass daily	Alcohol <10 g/day	Total amount of alcohol/day, e.g., beer, wine, spirits	1
**∑**			**11**

FFQ: Food Frequency Questionnaire; points were awarded for fulfilling the respective operationalization of a healthy diet based on,^32^ respectively, applying the same cutoffs as Voortman et al. (2017). For components of the diet score comprising several items (e.g., meat, dairy products), specific FFQ-items contributing to the score could vary between observations.

Participants provided sociodemographic and further information (education, net equivalence income in Euros, marital status, cognitive functioning, social network, self-rated health) during a structured face-to-face interview (baseline visit). Educational attainment was assessed according to CASMIN (Comparative Analysis of Social Mobility in Industrial Nations;^35^), comprising information on formal and vocational education and structured by three levels: low (e.g., early vocational qualifications); medium (e.g., high school diploma or equivalent), and high (e.g., university degree or higher professional qualification). Cognitive performance (Montreal Cognitive Assessment, MoCA;^36^ was assessed by trained study nurses during said baseline visit. Participants’ social network was measured using the 6 item-version of the Lubben Social Network-Scale (LSNS-6;^37^). The LSNS-6 assesses the number of friends and family members an individual can rely upon, asking, e.g., “How many relatives (friends) do you feel at ease with that you can talk about private matters?”, response options being: none (0 points), 1 (1 point), 2 (2 points), 3 or 4 (3 points), 5–8 (4 points), ≥9 (5 points). Answers are summed up, resulting in a score ranging from 0 to 30, with higher values indicating larger social networks. Self-rated health was measured using the EQ-VAS (EuroQol Visual Analogue Scale), asking participants to rate their current health status on a visual analogue scale with a range from 0 (worst imaginable health) to 100 (best imaginable health;^38^). We further assessed motivation for healthy eating using the item “How important is it to you to eat healthily?” with response options ranging from 0 (not at all) to 4 (very). Diet-specific self-efficacy was captured using an instrument developed by Schwarzer and Renner.^39^ It consisted of five items, which were each introduced with “I can stick to a healthy diet even…” (e.g.,: “if I have to start all over again several times until I succeed”; “if I initially don’t get much support”), response options ranging from 0 = very uncertain to 3 = very certain. Based on these questions, a mean score (range: 0–3) was calculated, with higher values indicating higher levels of self-efficacy.

Lastly, participants’ stage of health behavior change was assessed exemplarily for fruit and vegetable consumption and response options were operationalized in relation to the TTM. Participants were asked: “Do you eat at least five portions of fruit and vegetables daily?”. Response options were: “no, and I do not intend to” (precontemplation stage); “no, but I am thinking about it” (contemplation stage), “no, but I intend to do so” (preparation stage); “yes, but I find it hard” (action stage); “yes, and I find it easy” (maintenance stage). Participants were asked specifically about fruit and vegetable consumption to provide an anchor when asking about a healthy diet and to increase comparability of answers between participants. Further, this component of a healthy diet was chosen due to the overall high awareness of the importance of fruit and vegetable consumption for a healthy lifestyle.^40^

### Statistical analyses

Multivariable regression analyses were used to examine associations between sociodemographic, social, health and psychological factors with a healthy diet. Inspection of incomplete data revealed no evidence of systematically missing values; therefore, missing values were assumed to be missing at random (MAR) and were imputed using multiple imputation by chained equations, with predictive mean matching (PMM) applied for continuous variables (k = 10 nearest neighbors) and logistic regression with augmentation used for binary variables. The imputation model included all variables used in the analysis. We generated 50 imputed datasets with 500 iterations per dataset, following a burn-in of 5 iterations to ensure stability of the imputation algorithm. A random seed was set to allow reproducibility. Variables with no missing values (e.g., age, sex, and education) were registered as regular predictors to guide the imputation process. Imputations were performed at the item level of the food frequency questionnaire. Following imputation, the composite diet score was calculated. Regression analyses are based on the pooled results of 50 imputed datasets. Healthy eating was operationalized as a diet score across eleven dietary components. Distribution of values of the healthy diet score, assessed using histograms and skewness-kurtosis-tests, revealed no significant deviations from the normal distribution (*p *= 0.1135), therefore, linear regression models were calculated. The alpha-level was set at 0.05 (two-tailed). Additionally, logistic regressions were calculated to explore the association between sociodemographic, social, health, psychological factors, diet-specific self-efficacy and motivation for behavior change with eleven components of a healthy diet. To correct for multiple testing, Bonferroni correction was used, and the p-value was set at *p *< 0.005. Clustering of participants in general practices (n = 119; mean number of recruited participants per cluster: 14, IQR: 5) was accounted for through robust sandwich estimators. Lastly, we provide a descriptive analysis of stages of behavior change regarding a healthy diet in the baseline-sample of the AgeWell.de-study. All analyses were conducted in Stata 16.1 (StataCorp).

## Results

### Description of the sample

Of the 1030 participants from the baseline assessment of the AgeWell.de trial, 1001 (97.2%) had answered at least one of the items relevant for the healthy diet score and were included in our analyses. Participant characteristics (total sample, and stratified by sex; unimputed data) are described in Table 2.

**Table 2.** Description of participant characteristics.

	n	Total sample (n = 1001)	Men (n = 478)	Women (n = 523)	*p*
*Sociodemographic factors*					
Age, *M (SD)*	1001	69.0 (4.9)	68.7 ± 4.9	69.2 ± 4.9	0.101^a^
Education, *n (%)*	1001				**<0.001** ^b^
low		246 (24.6)	127 (26.6)	119 (22.8)	
medium		528 (52.8)	220 (46.0)	308 (58.9)	
high		227 (22.7)	131 (27.4)	96 (18.4)	
Equivalence income in Euros, *M (SD)*	930	1572 (846)	1655 (922)	1497 (763)	**0.004** ^a^
*Social factors*					
Married / cohabitating, *n (%)*	1001	645 (64.4)	369 (77.2)	276 (52.8)	**<0.001** ^b^
Social network (LSNS-6), *M (SD)*	999	2.9 (0.9)	2.8 (1.0)	2.9 (0.9)	**0.008** ^a^
*Health factors*					
Cognitive functioning (MoCA), *M (SD)*	999	24.6 (3.0)	24.1 (3.2)	25.0 (2.9)	**<0.001** ^a^
Self-rated health (EQ-VAS), *M (SD)*	998	76.3 (15.9)	77.0 (15.4)	76 (16.4)	0.216^a^
*Psychological factors*					
Motivation, *M (SD)*	987	3.3 (0.9)	3.2 (0.9)	3.4 (0.8)	**<0.001** ^a^
Diet-specific self-efficacy, *M (SD)*	940	1.9 (0.8)	1.8 (0.9)	2.0 (0.7)	**0.009** ^a^
*Healthy Diet*					
Healthy Diet Score, *M (SD)*	617	4.2 (1.7)	3.8 (1.6)	4.6 (1.6)	**<0**.**001**^a^
Vegetables ≥200 g/day, *n (%)*	915	184 (20.1)	56 (13.2)	128 (26.1)	**<0**.**001**^b^
Fruits ≥200 g/day, *n (%)*	961	453 (47.1)	188 (40.9)	265 (52.9)	**<0**.**001**^b^
Whole grains ≥90 g/day, *n (%)*	972	359 (36.9)	169 (36.4)	190 (37.4)	0.752^b^
Legumes ≥135 g/week, *n (%)*	978	199 (20.4)	96 (20.6)	103 (20.2)	0.877^b^
Nuts ≥15 g/day, *n (%)*	985	112 (11.4)	57 (12.2)	55 (10.6)	0.447^b^
Fish ≥100 g/week, *n (%)*	922	518 (56.2)	261 (59.1)	257 (53.5)	0.092^b^
Tea ≥ 450 ml/day, *n (%)*	953	61 (6.4)	34 (7.5)	27 (5.4)	0.202^b^
Dairy ≥350 g/day, *n (%)*	922	317 (34.4)	146 (33.3)	171 (35.3)	0.524^b^
Red and processed meat <300 g/week, *n (%)*	857	287 (33.5)	80 (19.3)	207 (46.8)	**<0**.**001**^b^
Sugar-containing beverages <150 ml/day, *n (%)*	949	858 (90.4)	400 (87.2)	458 (93.5)	**0**.**001**^b^
Alcohol <10 g/day, *n (%)*	862	556 (64.5)	207 (49.3)	349 (79.0)	**<0**.**001**^b^

EQ-VAS: EuroQol Visual Analogue Scale; Lubben Social Network Scale; M: Mean; MoCA: Montreal Cognitive Assessment; SD: standard deviation; ^a^t-test, ^b^χ² test. Boldface indicates statistical significance at the *p* < 0.05 level (unadjusted *p*-values).

Men and women differed regarding sociodemographic, social and health-related factors. Further, levels of motivation and diet-specific self-efficacy were higher in women. Responses to individual items and response categories for motivation and diet-specific self-efficacy are displayed in Supplemental Table 1. Median value of the healthy diet score was 4 (*IQR *= 2) in the total sample, with observed values ranging from 0 to 10 points (not tabulated; possible range: 0–11). Women revealed more favorable diets than men, indicated by the healthy diet score and adherence to recommendations for individual components of a healthy diet.

Including consumption of cheese and cream cheese in the assessment of dairy products might raise the risk of including foods high in saturated fat, possibly inducing risk of detrimental health effects. To account for this, we created a modified version of the healthy diet score, with dairy products excluding consumption of cheese and cream cheese. In the total sample, n = 281 (29.6%) met the recommended amount for consumption of dairy products (modified version; men: n = 122 (27.0%), women: n = 159 (31.9%)). Mean values (SD) of the modified healthy diet score were 4.2 (1.7) in the total sample, and 3.7 (1.6) for men, 4.6 (1.6) and women, respectively (*p *< 0.001; see Supplemental Table 2).

### Factors associated with a healthy diet

Table 3 describes the outcomes of the multivariable linear regression for factors associated with a healthy diet, as captured by the healthy diet score.

**Table 3.** Results of the linear regression of factors associated with a healthy diet (total score).

	b	SE	95% CI		*p*	
			Lower	Upper		
*Sociodemographic factors*						
Age	0.01	0.01	−0.01	0.04	0.209	
Female sex	0.64	0.12	0.41	0.88	**<0**.**001**	***
Education level medium	0.08	0.14	−0.19	0.35	0.563	
Education level high (ref.: low)	0.15	0.18	−0.21	0.51	0.411	
Income	−0.05	0.03	−0.11	0.01	0.113	
*Social factors*						
Married/cohabitating (ref.: single)	−0.15	0.12	−0.38	0.08	0.186	
Social network	0.06	0.06	−0.06	0.17	0.345	
*Health factors*						
Cognitive functioning	0.01	0.02	−0.03	0.05	0.543	
Self-rated health	0.00	0.00	0.00	0.01	0.460	
*Psychological factors*						
Motivation	0.22	0.06	0.10	0.34	**0**.**001**	**
Diet-specific self-efficacy	0.33	0.06	0.20	0.46	**<0**.**001**	***

SE: standard error; CI: confidence interval; **p < 0.01, ***p < 0.001. Boldface indicates statistical significance at the *p* < 0.05 level.

Female sex (b = 0.64, 95% CI: 0.41, 0.88) was linked to consumption of a healthy diet, as assessed by the healthy diet score. Moreover, higher levels of motivation for healthy eating (b = 0.22, 95% CI: 0.10, 0.34) and diet-specific self-efficacy (b = 0.33, 95% CI; 0.20, 0.46) were associated with a healthy diet.

Factors linked to consumption of single items comprised by the healthy diet score are given in Table 4.

**Table 4.** Results of logistic regressions of factors associated with healthy diet components.

	OR (95% CI) for vegetables ≥ 200 g/day	OR (95% CI) for fruit ≥ 200 g/day	OR (95% CI) for whole grain products ≥ 90 g/day	OR (95% CI) for legumes ≥ 135 g/week	OR (95% CI) for nuts ≥ 15 g/day	OR (95% CI) for fish ≥ 100 g/week
*Sociodemographic factors*						
Age	0.97 (0.94, 1.00)	1.04 (1.01, 1.06)**	1.01 (0.98, 1.04)	0.99 (0.96, 1.03)	1.02 (0.99, 1.06)	1.00 (0.97, 1.03)
Female sex	2.42 (1.73, 3.38)***^†^	1.45 (1.10, 1.92)**	0.93 (0.69, 1.27)	0.91 (0.66, 1.26)	0.84 (0.56, 1.27)	0.79 (0.58, 1.06)
Education medium	1.44 (0.92, 2.26)	1.43 (1.03, 1.99)*	0.95 (0.69, 1.30)	1.04 (0.69, 1.57)	1.01 (0.61, 1.66)	1.11 (0.79, 1.55)
Education high (ref.: low)	1.21 (0.69, 2.14)	1.72 (1.12, 2.66)*	0.82 (0.54, 1.26)	1.30 (0.80, 2.12)	0.97 (0.56, 1.67)	1.22 (0.81, 1.84)
Income	1.03 (0.93, 1.15)	0.95 (0.86, 1.05)	0.95 (0.86, 1.06)	0.93 (0.83, 1.03)	1.10 (1.00, 1.20)*	0.98 (0.91, 1.06)
*Social factors*						
Marital status (ref.: single)	1.37 (0.94, 1.99)	0.84 (0.65, 1.10)	0.85 (0.63, 1.15)	1.03 (0.72, 1.47)	1.19 (0.79, 1.81)	1.16 (0.89, 1.52)
Social network	1.07 (0.88, 1.29)	1.13 (0.99, 1.30)	1.03 (0.89, 1.19)	1.22 (1.00, 1.49)*	1.03 (0.81, 1.31)	1.18 (1.01, 1.39)*
*Health factors*						
Cognitive functioning	1.03 (0.97, 1.09)	1.01 (0.97, 1.06)	1.01 (0.95, 1.07)	1.00 (0.94, 1.05)	1.00 (0.93, 1.07)	1.04 (0.99, 1.09)
Self-rated health	1.00 (0.99, 1.01)	1.00 (0.99, 1.01)	1.00 (0.99, 1.01)	1.00 (0.99, 1.01)	1.00 (0.99, 1.01)	1.00 (0.99, 1.01)
*Psychological factors*						
Motivation	1.25 (0.98, 1.59)	1.28 (1.09, 1.50)***^†^	1.05 (0.88, 1.25)	1.07 (0.88, 1.31)	1.28 (0.97, 1.68)	0.96 (0.83, 1.11)
Diet-specific self-efficacy	1.54 (1.22, 1.95)***^†^	1.24 (1.02, 1.51)*	1.17 (0.96, 1.43)	1.20 (0.95, 1.51)	1.50 (1.12, 2.02)**	1.15 (0.96, 1.38)

CI: confidence interval; OR: odds ratio. *p < 0.05, **p < 0.01, ***p < 0.001 (unadjusted p-values). ^†^*p *< 0.005 (Bonferroni-adjusted p-value).

**Table:** 

	OR (95% CI) for tea ≥ 450 ml/day	OR (95% CI) for dairy ≥ 350 g/day	OR (95% CI) for red and processed meat < 300 g/week	OR (95% CI) for sugar-containing beverages < 150 ml/day	OR (95% CI) for alcohol < 10 g/day
*Sociodemographic factors*					
Age	1.02 (0.96, 1.08)	0.99 (0.96, 1.02)	1.02 (0.99, 1.05)	1.05 (1.00, 1.09)*	1.00 (0.97, 1.04)
Female sex	0.68 (0.34, 1.37)	1.00 (0.75, 1.33)	3.04 (2.19, 4.23)***^†^	1.75 (1.07, 2.85)*	3.93 (2.83, 5.46)***^†^
Education medium	0.69 (0.36, 1.33)	1.04 (0.73, 1.49)	0.79 (0.52, 1.18)	1.01 (0.57, 1.78)	0.95 (0.66, 1.38)
Education high (ref.: low)	0.57 (0.25, 1.30)	1.36 (0.86, 2.16)	1.00 (0.62, 1.62)	1.18 (0.54, 2.57)	0.67 (0.41, 1.10)
Income	1.26 (1.12, 1.41)***^†^	0.92 (0.84, 1.01)	0.97 (0.89, 1.05)	1.04 (0.91, 1.20)	0.87 (0.80, 0.94)**
*Social factors*					
Marital status (ref.: single)	0.99 (0.51, 1.91)	0.71 (0.51, 0.98)*	0.63 (0.46, 0.87)**	0.94 (0.58, 1.51)	1.00 (0.71, 1.41)
Social network	0.95 (0.73, 1.23)	1–04 (0.91, 1.19)	0.89 (0.75, 1.04)	1.08 (0.84, 1.41)	0.76 (0.66, 0.88)***^†^
*Health factors*					
Cognitive functioning	1.01 (0.91, 1.12)	0.99 (0.94, 1.03)	1.01 (0.96, 1.06)	1.04 (0.96, 1.12)	0.96 (0.91, 1.02)
Self-rated health	0.99 (0.97, 1.01)	1.00 (0.99, 1.01)	1.01 (1.00, 1.02)	1.00 (0.98, 1.01)	1.01 (1.00, 1.02)
*Psychological factors*					
Motivation	1.57 (1.00, 2.47)	1.11 (0.94, 1.31)	1.18 (0.98, 1.41)	1.19 (0.93, 1.54)	1.08 (0.91, 1.30)
Diet-specific self-efficacy	1.27 (0.90, 1.79)	1.07 (0.90, 1.26)	1.20 (0.99, 1.47)	1.28 (0.95, 1.73)	1.00 (0.81, 1.24)

CI: confidence interval; OR: odds ratio. Unadjusted p-values: *p < 0.05, **p < 0.01, ***p < 0.001. Bonferroni-adjusted p-value: ^†^*p *< 0.005.

To assess the robustness of our findings, sensitivity analyses using the original data without imputations were conducted, both for the healthy diet score (Supplemental Table 3) and its respective components (Supplemental Table 4). Regression results based on the unimputed data were very similar, supporting the robustness of the findings (see Supplemental Material). We further assessed potential influence of missing data, applying a pattern-mixture model (outcome: healthy diet score). The respective model included an indicator of missing values (1 if any values on components of the healthy diet score were missing, 0 if no values were missing) and interaction terms to detect whether associations of age, sex or education depended on data being missing. Results were highly comparable to the original model, supporting the assumption of data being missing at random (Supplemental Table 6).

Regression results for the modified healthy diet score, excluding consumption of cheese and cream cheese, are provided in Supplemental Table 7. Results remained highly comparable, arguing for robustness of our findings.

### Missing data

Men in our sample had more missing values on vegetable consumption (*p *= 0.007). For participants with lower levels of education, more values were missing for income (*p *= 0.048), consumption of vegetables (*p *= 0.001), legumes (*p *< 0.001), nuts (*p *= 0.018), fish (*p *= 0.001), dairy products (*p *= 0.001), red/processed meat (*p *= 0.020), sugar-containing beverages (*p *= 0.025), and the healthy diet score (*p *< 0.001). Older age was linked to higher likelihood of missing values on diet-specific self-efficacy (*p *= 0.018).

Following established recommendations,^41^ we conducted a quantitative bias analysis to verify robustness of results against possible violations of the MAR-assumption, applying a shift (δ = ± 0.2) to the imputed values of the healthy diet score in each of the imputed datasets. The delta-adjusted analysis produced estimates consistent with the primary findings (Supplemental Table 5), suggesting that the observed associations are robust to moderate deviations from the MAR assumption.

### Stages of behavior change

Distribution of participants across stages of health behavior change according to the TTM are described in Table 5.

**Table 5.** Distribution of participants across stages of health behavior change.

Do you eat at least five portions of fruits and vegetables every day?		Stage of health behavior change	Number (%) of participants
*No, …*			
	*and I do not intend to.*	Precontemplation	208 (21.2)
	*but I think about it.*	Contemplation	211 (21.5)
	*but I intend to do so.*	Preparation	77 (7.8)
*Yes, and …*			
	*I find it difficult.*	Action	44 (4.5)
	*I find it easy.*	Maintenance	444 (45.2)
			Σ = 983

## Discussion

The first aim of the present study was to investigate sociodemographic, social, health and psychological factors associated with a healthy diet in older adults at increased risk for dementia. The second aim was to describe the distribution across the stages of health behavior change towards a healthy diet according to the transtheoretical model (TTM;^24,25^). Using a comprehensive score capturing a healthy diet and single elements of the respective score as outcomes, we assessed factors linked to a healthy diet in participants of AgeWell.de, a multidomain lifestyle-based intervention to preserve cognitive function. Overall, our sample revealed room for improvement regarding a healthy diet, with an average score of 4.2 out of 11 possible points. Regarding stages of behavior change, 45% had reached the latest stage - the maintenance stage, in which participants reported to consume the sufficient amount of fruits and vegetables and found it easy to do so. However, about half of participants did not adhere to this dietary guideline.

Women consumed healthier diets, assessed by the healthy diet score, corroborating findings from other studies reporting healthier dietary patterns in women.^17,18,42^ Traditional gender roles, where duties like cooking and being responsible for the nutrition of families are more often deemed a female responsibility, might partly explain this association, but also exposure to knowledge about nutrition: Over the life course, women enter settings in which people learn about a healthy diet (e.g., healthcare settings, grocery stores) more often than men. This may constitute an advantage regarding knowledge about healthy eating.^42^ Higher levels of concern about weight gain, higher dissatisfaction with being overweight^43,44^ overall higher levels of health consciousness and participation in health-promoting activities^45,46^ in women might further explain the observed sex differences in our study.

Findings regarding age and a healthy diet are inconsistent, with some studies reporting no association between age and a Mediterranean diet^47^ or consumption of fruit, vegetables and legumes,^48^ while others found negative associations of age and consumption of vegetables, fruit and grains.^49^ In a recent study on older German adults, age was negatively linked to dietary risk behavior (insufficient consumption of vegetables/fruit, whole grain and dairy products) in men only.^50^

As levels of education and income were overall rather high in our sample, the non-significant associations with a healthy diet and low explanatory power for individual dietary components might be due to limited variability in the respective variables. While some studies reported positive associations between higher education and fruit and vegetable consumption,^51,52^ others found no association^53^ or negative associations.^54^ Similar to our findings regarding socioeconomic and psychological factors linked to healthy eating, a study in older German adults reported that a healthy diet was predicted by attitudes and beliefs, but not by socioeconomic status.^55^ Neither cognitive performance nor self-rated health explained differences in eating behavior in our sample. Participants in AgeWell.de were, on average, only minimally impaired regarding health and cognition, suggesting that links between cognitive performance/self-rated health and dietary habits might become apparent only at more severe levels of impairment.

Married/cohabitating participants revealed less favorable eating behaviors regarding consumption of dairy products and red meat, in line with two studies from France suggesting that living in a partnership or cohabitation is associated with unhealthier diets in older adults.^19,20^ Larger social networks, which are commonly linked to healthier eating habits, were associated with higher consumption of fish and legumes, however, people with larger social networks also consumed more alcohol, which is in line with previous findings.^56^ People with larger social networks might participate in social gatherings where alcohol is consumed more often, which might explain this finding.

Higher levels of motivation for a healthy diet and diet-specific self-efficacy were linked to higher healthy diet scores, corroborating previous work reporting a strong influence of self-efficacy^57^ and motivation^58^ on intake of fruit and vegetables in adult populations. Similar results were also reported in a Dutch study among adults aged 40–79 years, identifying perceived behavioral control as the most important determinant of several health behaviors, including a healthy diet.^59^ This underlines the importance of self-efficacy and motivation in designing and implementing interventions for healthy aging and dementia risk reduction. Tailoring interventions to participants’ current level of self-efficacy and stage of behavior change might increase effectiveness of lifestyle interventions.^22,58^ This might include specific intervention components targeting participants’ self-efficacy and control beliefs regarding lifestyle changes.

For participants in the pre-contemplation, contemplation or preparation stage of behavior change (50.5% of participants in our sample), raising awareness about the role of nutrition and promoting positive thoughts about healthy dietary changes might be helpful to change individuals’ risk perception and promote cues to action, resulting in transition to a later stage of behavior change.^60^ Respective measures for interventions could include, for example, food diaries or digital measures to track intake of specific foods to increase awareness and motivation.^61^ Supporting individuals in setting realistic goals^62^ and promoting coping skills for dealing with setbacks and relapses to old behaviors^63^ might further aid in motivating participants towards healthy dietary changes. This might be especially relevant for persons in the action-stage, who have already introduced healthy behavior changes but report difficulties in maintaining them (4.5% in our sample). On the other hand, 45% of the sample had reached the latest stage of change (maintenance stage). FFQ-data for consumption of fruit and vegetables (Table 2) suggest that consumption of these two food groups differed in our sample, with 47.1% of participants eating the recommended amount of fruit, but only 20.1% eating the recommended amount of vegetables daily. This might indicate that having reached the action- or maintenance-stage is mostly driven by consumption of fruit and less so by vegetable intake. It has to be pointed out that these figures might be only partially generalizable to the older public, since all participants in our study had agreed to participate in a lifestyle intervention including optimization of nutrition. Baseline motivation for behavior change may therefore have been higher than what could be expected in population-based samples. Further, the stages of change were assessed by asking about participants’ motivation for consuming specific components of a healthy diet, i.e., fruit and vegetables. This approach was chosen to provide an anchor for participants and increase comparability of answers across the sample. Results may have differed when using a more general assessment of a healthy diet, and participants’ stage of behavior change may have varied between different components of a healthy diet.

Our data revealed lower levels of diet-specific self-efficacy and motivation for healthy eating in men. While some earlier studies^64,65^ and an investigation from Shandong, China^66^ reported higher levels of self-efficacy in men, Emanuel and colleagues reported more favorable attitudes and higher levels of perceived behavioral control regarding fruit and vegetable consumption in women.^67^ However, research on gender differences in (health-related) self-efficacy in older adults is scarce. Despite changes in traditional gender roles in the last decades, the topic of nutrition, cooking etc. is still considered a traditionally female domain (see above), which may explain women's higher levels of motivation and self-efficacy when engaging with a healthy diet. This suggests that lifestyle interventions including a dietary component should put a special emphasis on increasing (diet-specific) self-efficacy and motivation in older men to maximize effectiveness.

### Strengths and limitations

Strengths of our study include a large sample size, and a comprehensive assessment of a healthy diet covering a variety of foods/food groups, thereby exceeding previous investigations focusing primarily on fruit- and vegetable intake. These findings might be useful to inform future interventions targeting optimization of nutrition in older adults, particularly in those with an increased dementia risk. Further, we were able to assess a comprehensive set of covariates and their associations with a healthy diet. By investigating data from older adults at increased risk for dementia, we provide insights potentially useful for the design and implementation of future trials targeting cognitive decline/dementia risk reduction and other aspects of healthy aging.

However, using data from an at-risk sample for dementia also constitutes a limitation of our study, as results might only partially be generalizable to the general older public. Nevertheless, since older adults at increased risk for dementia constitute a highly relevant target group for measures of health promotion and primary prevention, our results may be of great use for future trials in the field. Second, questions regarding stages of health behavior change according to TTM focused on fruit and vegetable consumption, while our main outcome was a composite healthy diet score. Capturing a person's eating behavior in detail and validly in a study is challenging. Due to self-reported information on food consumption, social desirability may have affected our findings. Considering the generally moderate values of the healthy diet score, however, this potential effect is deemed negligible. Moreover, although dairy products are typically considered a part of a healthy diet, consumption of dairy products following the approach by Voortman and colleagues^32^ included foods potentially high in saturated fat (cheese, cream cheese), inducing risk of detrimental health effects. While neither the Dutch nor German guidelines on a healthy diet make statements regarding a preference of low-fat over whole-fat dairy products, this approach runs the risk of overlooking potentially detrimental effects of high intake of saturated fats. Using more nuanced assessments of dietary intake that take into account amounts of (un)saturated fat intake might provide valuable results in future studies. Inspection of our data revealed a high consumption of cream cheese (>90 g/daily) or cheese (>120 g/daily) in less than 2% of the sample. To account for this, we conducted sensitivity analyses, using a modified healthy diet score, excluding consumption of cheese and cream cheese, which did not significantly alter results of regression analyses for the healthy diet score. Amounts of missing data on components of the healthy diet score were rather high in our sample, which was addressed by multiple imputation. This approach relies on the assumption of data being missing at random. While we cannot fully rule out that missingness depended on potentially unobserved characteristics, we conducted several sensitivity analyses (including a quantitative bias analyses and pattern-mixture-models) to ensure that our findings are robust when assuming slight deviations from the MAR-assumption. Finally, the FFQ applied in the AgeWell.de-study did not allow for the assessment of three of the 14 components included in the original healthy diet score by Voortman and colleagues,^32^ namely sodium intake, proportion of whole grain products of total grain products and use of unsaturated fats and oils (≥ 50% of total fats). Including these information in future trials might provide even more robust results. However, we were able to apply a comprehensive score including a variety of foods shown to be essential for a healthy diet. We are therefore confident that our findings still provide a valuable contribution to the field.

### Conclusion

Our study highlights the link between older adults’ self-efficacy and motivation for healthy eating and consumption of a healthy diet. Interventions aimed at healthy aging and dementia risk reduction might benefit not only from providing targeted strategies to promote healthy eating in older adults, but also from including measures to promote participants’ self-efficacy and motivation. Respective approaches might empower a larger share of older adults to engage in healthy lifestyles and thereby increase reach and effectiveness of interventions for healthy aging.

## Supplemental Material

sj-docx-1-alz-10.1177_13872877251330296 - Supplemental material for Factors associated with a healthy diet and willingness to change dietary behavior in older adults at increased risk of dementia Supplemental material, sj-docx-1-alz-10.1177_13872877251330296 for Factors associated with a healthy diet and willingness to change dietary behavior in older adults at increased risk of dementia by Iris Blotenberg, Andrea E Zülke, Melanie Luppa, Felix Wittmann, Thomas Fankhänel, Solveig Weise, Juliane Döhring, Catharina Escales, Robert P Kosilek, Irina Michel, Christian Brettschneider, Anke Oey, Birgitt Wiese, Jochen Gensichen, Hans-Helmut König, Thomas Frese, Hanna Kaduszkiewicz, Wolfgang Hoffmann, Steffi G Riedel-Heller and René Thyrian in Journal of Alzheimer's Disease
