# Supplementary material for: Factors associated with a healthy diet and willingness to change dietary behavior in older adults at increased risk of dementia
Source: J Alzheimers Dis. 2025 Apr 15;105(2):634–45. doi: 10.1177/13872877251330296 (PMC12231830; doi:10.1177/13872877251330296)
Supplement: sj-docx-1-alz-10.1177_13872877251330296 - Supplemental material for Factors associated with a healthy diet and willingness to change dietary behavior in older adults at increased risk of dementia [file sj-docx-1-alz-10.1177_13872877251330296.docx]

**Supplemental Material**

**Factors associated with a healthy diet and willingness to change dietary behavior in older adults at increased risk of dementia**

**Supplemental Table 1.** Participant responses to individual items and response categories assessing motivation and diet-specific self-efficacy

|  | | n | | Total sample  (n = 1,001) | | Men  (n = 478) | | Women  (n = 523) | | *p* | |  |  |  |
| --- | --- | --- | --- | --- | --- | --- | --- | --- | --- | --- | --- | --- | --- | --- |
| ***Psychological factors*** | |  | |  | |  | |  | |  | |  |  |  |
| **Motivation**, M (SD) | | 987 | | 3.3 (0.9) | | 3.2 (0.9) | | 3.4 (0.8) | | **<0.001**^a^ | |  |  |  |
| “How important is it to you to eat healthily?” | | | | | | | | | | | | |  |  |
| Not at all, *n (%)* | |  | | 11 (1.1) | | 10 (2.1) | | 1 (0.2) | | **<0.001**^b^ | |  |  |  |
|  | |  | | 12 (1.2) | | 4 (0.9) | | 8 (1.6) | |  | |  |  |  |
|  | |  | | 196 (19.9) | | 116 (24.6) | | 80 (15.5) | |  | |  |  |  |
|  | |  | | 185 (18.7) | | 85 (18.1) | | 100 (19.4) | |  | |  |  |  |
| Very, *n (%)* | |  | | 583 (59.1) | | 256 (54.4) | | 327 (63.4) | |  | |  |  |  |
| **Diet-specific self-efficacy**, ***M (SD)*** | | 940 | | 1.9 (0.8) | | 1.8 (0.9) | | 2.0 (0.7) | | **0.009**^a^ | |  |  |  |
| **Individual items, *n (%)*** | |  | |  | |  | |  | |  | |  |  |  |
| “There are sometimes obstacles that can make it difficult to change your diet. How confident are you that you could overcome the following obstacles?  I can stick to a healthy diet even... | | | | | | | | | | | | |  |  |
| … if it takes me a long time for the new diet to become part of my everyday life. | | | | | | | | | | | | |  |  |
| Very uncertain, *n (%)* | | 940 | | 68 (7.2) | | 49 (11.0) | | 19 (3.9) | | **<0.001**^b^ | |  |  |  |
| Rather uncertain, *n (%)* | |  | | 230 (24.5) | | 99 (22.2) | | 131 (26.5) | |  | |  |  |  |
| Rather certain, *n (%)* | |  | | 376 (40.0) | | 176 (39.5) | | 200 (40.5) | |  | |  |  |  |
| Very certain, *n (%)* | |  | | 266 (28.3) | | 122 (27.4) | | 144 (29.2) | |  | |  |  |  |
| …if I have to start all over again several times until I succeed | | | | | | | | | | | | |  |  |
| Very uncertain, *n (%)* | | 939 | | 73 (7.8) | | 51 (11.5) | | 22 (4.5) | | **0.001**^b^ | |  |  |  |
| Rather uncertain, *n (%)* | |  | | 205 (21.8) | | 94 (21.1) | | 111 (22.5) | |  | |  |  |  |
| Rather certain, *n (%)* | |  | | 376 (40.0) | | 165 (37.1) | | 211 (42.7) | |  | |  |  |  |
| Very certain, *n (%)* | |  | | 285 (30.4) | | 135 (30.3) | | 150 (30.4) | |  | |  |  |  |
| …if I have to rethink my diet as a result. | | | | | | | | | | | | |  |  |
| Very uncertain, *n (%)* | |  | | 86 (9.2) | | 59 (13.3) | | 27 (5.5) | | **<0.001**^b^ | |  |  |  |
| Rather uncertain, *n (%)* | |  | | 234 (25.0) | | 102 (22.9) | | 132 (26.8) | |  | |  |  |  |
| Rather certain, *n (%)* | |  | | 369 (39.4) | | 165 (37.1) | | 204 (41.5) | |  | |  |  |  |
| Very certain, *n (%)* | |  | | 248 (26.5) | | 119 (26.7) | | 129 (26.2) | |  | |  |  |  |
| …if I initially don’t get much support | | | | | | | | | | | | |  |  |
| Very uncertain, *n (%)* | |  | | 84 (8.9) | | 60 (13.5) | | 24 (4.9) | | **<0.001**^b^ | |  |  |  |
| Rather uncertain, *n (%)* | |  | | 222 (23.6) | | 105 (23.5) | | 117 (23.7) | |  | |  |  |  |
| Rather certain, *n (%)* | |  | | 361 (38.4) | | 157 (35.2) | | 204 (41.3) | |  | |  |  |  |
| Very certain, *n (%)* | |  | | 273 (29.0) | | 124 (27.8) | | 149 (30.2) | |  | |  |  |  |
| ...if I have to plan it at the beginning. | | | | | | | | | | | | |  |  |
| Very uncertain, *n (%)* | |  | | 75 (8.0) | | 50 (11.2) | | 25 (5.1) | | **0.003**^b^ | |  |  |  |
| Rather uncertain, *n (%)* | |  | | 205 (21.8) | | 101 (22.7) | | 104 (21.1) | |  | |  |  |  |
| Rather certain, *n (%)* | |  | | 385 (41.0) | | 166 (37.2) | | 219 (44.3) | |  | |  |  |  |
| Very certain, *n (%)* | |  | | 275 (29.3) | | 129 (28.9) | | 146 (29.6) | |  | |  |  |  |
| M: mean; SD: standard deviation; ^a^ t-test, ^b^ χ² test. unadjusted p-values. | | | | | | | | | | | | |  |  |
| **Supplemental Table 2.** Description of participant characteristics, modified assessment of a healthy diet | | | | | | | | | | | | | | |
|  | | | n | | Total sample  (n = 1,001) | | Men  (n = 478) | | Women  (n = 523) | | *p* | | |  |
| ***Sociodemographic factors*** | | |  | |  | |  | |  | |  | | |  |
| Age, *M (SD)* | | | 1,001 | | 69.0 (4.9) | | 68.7±4.9 | | 69.2±4.9 | | 0.101^a^ | | |  |
| Education, *n (%)* | | | 1,001 | |  | |  | |  | | <0.001^b^ | | |  |
|  | low | |  | | 246 (24.6) | | 127 (26.6) | | 119 (22.8) | |  | | |  |
|  | medium | |  | | 528 (52.8) | | 220 (46.0) | | 308 (58.9) | |  | | |  |
|  | high | |  | | 227 (22.7) | | 131 (27.4) | | 96 (18.4) | |  | | |  |
| Equivalence income in Euros,  *M (SD)* | | | 930 | | 1,572 (846) | | 1,655 (922) | | 1,497 (763) | | 0.004^a^ | | |  |
| ***Social factors*** | | |  | |  | |  | |  | |  | | |  |
| Married / cohabitating, *n (%)* | | | 1,001 | | 645 (64.4) | | 369 (77.2) | | 276 (52.8) | | <0.001^b^ | | |  |
| Social network (LSNS-6),  *M (SD)* | | | 999 | | 2.9 (0.9) | | 2.8 (1.0) | | 2.9 (0.9) | | 0.008^a^ | | |  |
| ***Health factors*** | | |  | |  | |  | |  | |  | | |  |
| Cognitive functioning (MoCA), *M (SD)* | | | 999 | | 24.6 (3.0) | | 24.1 (3.2) | | 25.0 (2.9) | | <0.001^a^ | | |  |
| Self-rated health (EQ-VAS), *M (SD)* | | | 998 | | 76.3 (15.9) | | 77.0 (15.4) | | 76 (16.4) | | 0.216^a^ | | |  |
| ***Psychological factors*** | | |  | |  | |  | |  | |  | | |  |
| Motivation, *M (SD)* | | | 987 | | 3.3 (0.9) | | 3.2 (0.9) | | 3.4 (0.8) | | <0.001^a^ | | |  |
| Diet-specific self-efficacy, *M (SD)* | | | 940 | | 1.9 (0.8) | | 1.8 (0.9) | | 2.0 (0.7) | | 0.009^a^ | | |  |
| ***Healthy Diet*** | | |  | |  | |  | |  | |  | | |  |
|  | Healthy Diet Score, *M (SD)* | | 627 | | 4.2 (1.7) | | 3.7 (1.6) | | 4.6 (1.6) | | <0.001^a^ | | |  |
|  | Vegetables ≥200 g/day, *n (%)* | | 915 | | 184 (20.1) | | 56 (13.2) | | 128 (26.1) | | <0.001^b^ | | |  |
|  | Fruits ≥200 g/day, *n (%)* | | 961 | | 453 (47.1) | | 188 (40.9) | | 265 (52.9) | | <0.001^b^ | | |  |
|  | Whole grains ≥90 g/day, *n (%)* | | 972 | | 359 (36.9) | | 169 (36.4) | | 190 (37.4) | | 0.752^b^ | | |  |
|  | Legumes ≥135 g/week, *n (%)* | | 978 | | 199 (20.4) | | 96 (20.6) | | 103 (20.2) | | 0.877^b^ | | |  |
|  | Nuts ≥15 g/day, *n (%)* | | 985 | | 112 (11.4) | | 57 (12.2) | | 55 (10.6) | | 0.447^b^ | | |  |
|  | Fish ≥100 g/week, *n (%)* | | 922 | | 518 (56.2) | | 261 (59.1) | | 257 (53.5) | | 0.092 ^b^ | | |  |
|  | Tea ≥ 450 ml/day, *n (%)* | | 953 | | 61 (6.4) | | 34 (7.5) | | 27 (5.4) | | 0.202^b^ | | |  |
|  | Dairy ≥350 g/day, *n (%)* | | 950 | | 281 (29.6) | | 122 (27.0) | | 159 (31.9) | | 0.096 | | |  |
|  | Red and processed meat <300 g/week, *n (%)* | | 857 | | 287 (33.5) | | 80 (19.3) | | 207 (46.8) | | <0.001^b^ | | |  |
|  | Sugar-containing beverages <150 ml/day, *n (%)* | | 949 | | 858 (90.4) | | 400 (87.2) | | 458 (93.5) | | 0.001^b^ | | |  |
|  | Alcohol <10 g/day, *n (%)* | | 862 | | 556 (64.5) | | 207 (49.3) | | 349 (79.0) | | <0.001^b^ | | |  |
| EQ-VAS: EuroQol Visual Analogue Scale*,* Lubben Social Network Scale; M: Mean; MoCA: Montreal Cognitive Assessment; SD: standard deviation; ^a^ t-test, ^b^ χ² test. unadjusted p-values. Assessment of dairy products excluding consumption of cheese and cream cheese. | | | | | | | | | | | | | | |

| **Supplemental Table 3.** Results of the linear regression of factors associated with a healthy diet based on unimputed data (total score). | | | | | | |  |
| --- | --- | --- | --- | --- | --- | --- | --- |
|  | b | SE | 95% CI | | *p* |  | |
|  |  |  | Lower | Upper |  |  | |
| ***Sociodemographic factors*** |  |  |  |  |  |  | |
| Age | 0.02 | 0.02 | -0.02 | 0.05 | 0.333 |  | |
| Female sex | 0.42 | 0.16 | 0.09 | 0.74 | 0.012 | * | |
| Education level medium | 0.09 | 0.16 | -0.22 | 0.40 | 0.574 |  | |
| Education level high (ref.: low) | 0.22 | 0.25 | -0.27 | 0.72 | 0.375 |  | |
| Income | -0.03 | 0.04 | -0.11 | 0.06 | 0.521 |  | |
| ***Social factors*** |  |  |  |  |  |  | |
| Married/cohabitating (ref.: single) | -0.23 | 0.13 | -0.49 | 0.03 | 0.088 |  | |
| Social network | 0.00 | 0.07 | -0.15 | 0.15 | 0.989 |  | |
| ***Health factors*** |  |  |  |  |  |  | |
| Cognitive functioning | 0.06 | 0.03 | 0.01 | 0.11 | 0.034 | * | |
| Self-rated health | 0.00 | 0.00 | -0.01 | 0.01 | 0.784 |  | |
| ***Psychological factors*** |  |  |  |  |  |  | |
| Motivation | 0.25 | 0.08 | 0.09 | 0.42 | 0.003 | ***^†^ | |
| Diet-specific self-efficacy | 0.26 | 0.09 | 0.07 | 0.44 | 0.008 | ** | |

| **Supplemental Table 4.** Results of logistic regressions of factors associated with healthy diet components based on unimputed data. | | | | | | |
| --- | --- | --- | --- | --- | --- | --- |
|  | OR (95% CI) for vegetables  ≥ 200 g/day | OR (95% CI) for fruit ≥ 200 g/day | OR (95% CI) for whole grain products  ≥ 90 g/day | OR (95% CI) for legumes  ≥ 135 g/week | OR (95% CI) for nuts ≥ 15 g/day | OR (95% CI) for fish  ≥ 100 g/week |
| ***Sociodemographic factors*** |  |  |  |  |  |  |
| Age | 0.96 (0.92, 1.01) | 1.02 (0.99, 1.06) | 1.00 (0.96, 1.05) | 1.02 (0.96, 1.07) | 1.04 (0.98, 1.11) | 1.02 (0.98, 1.06) |
| Female sex | 2.29 (1.51, 3.47)***^†^ | 1.42 (1.01, 2.01)* | 0.88 (0.59, 1.32) | 0.91 (0.54, 1.54) | 0.82 (0.46, 1.45) | 0.96 (0.70, 1.34) |
| Education medium | 1.25 (0.69, 2.28) | 1.41 0.88, 2.26) | 0.84 (0.58, 1.23) | 1.11 (0.65, 1.92) | 1.12 (0.50, 2.54) | 1.15 (0.76, 1.73) |
| Education high (ref.: low) | 1.16 (0.56. 2.39) | 1.69 (0.87, 3.31) | 0.99 (0.58, 1.70) | 1.56 (0.75, 3.20) | 1.69 (0.69, 4.14) | 1.30 (0.75, 2.24) |
| Income | 1.12 (0.97, 1.30) | 0.97 (0.85, 1.10) | 0.84 (0.74, 0.96)* | 0.99 (0.88, 1.11) | 1.14 (1.02, 1.28)* | 1.05 (0.97, 1.13) |
| ***Social factors*** |  |  |  |  |  |  |
| Marital status (ref.: single) | 1.03 (0.60, 1.78) | 0.73 (0.52, 1.02) | 1.15 (0.76, 1.73) | 1.12 (0.68, 1.87) | 1.12 (0.63, 2.00) | 1.30 (0.91, 1.86) |
| Social network | 1.09 (0.84, 1.41) | 1.07 (0.89, 1.29) | 0.97 (0.80, 1.18) | 1.28 (1.00, 1.63)* | 0.83 (0.59, 1.17) | 1.08 (0.89, 1.32) |
| ***Health factors*** |  |  |  |  |  |  |
| Cognitive functioning | 1.10 (1.01, 1.19)* | 1.05 (0.99, 1.11) | 1.02 (0.96, 1.09) | 1.03 (0.96, 1.11) | 1.03 (0.94, 1.12) | 1.06 (0.99, 1.13) |
| Self-rated health | 1.00 (0.99, 1.01) | 1.01 (1.00, 1.02) | 1.00 (0.99, 1.01) | 0.99 (0.98, 1.00)* | 1.00 (0.98, 1.01) | 1.00 (0.99, 1.01) |
| ***Psychological factors*** |  |  |  |  |  |  |
| Motivation | 1.24 (0.95, 1.62) | 1.20 (0.97, 1.49) | 0.95 (0.76, 1.20) | 1.08 (0.80, 1.47) | 1.35 (0.95, 1.92) | 1.06 (0.88, 1.27) |
| Diet-specific self-efficacy | 1.27 (0.90, 1.81) | 1.31 (0.98, 1.75) | 1.21 (0.92, 1.59) | 1.24 (0.90, 1.70) | 1.30 (0.96, 1.75) | 1.16 (0.93, 1.45) |
|  |  |  |  |  |  |  |
| CI: confidence interval; OR: odds ratio. **p* < 0.05, ***p* < 0.01, ****p* < 0.001 (unadjusted p-values). ^†^*p* < 0.005 (Bonferroni-adjusted p-value). | | | | | | |

| **Supplemental Table 4 (continued).** Results of logistic regressions of factors associated with healthy diet components based on unimputed data. | | | | | |
| --- | --- | --- | --- | --- | --- |
|  | OR (95% CI) for tea  ≥ 450 ml/day | OR (95% CI) for dairy  ≥ 350 g/day | OR (95% CI) for red and processed meat  < 300 g/week | OR (95% CI) for sugar-containing beverages  < 150 ml/day | OR (95% CI) for alcohol  < 10 g/day |
| ***Sociodemographic factors*** |  |  |  |  |  |
| Age | 0.99 (0.91, 1.08) | 0.98 (0.95, 1.02) | 1.01 (0.98, 1.05) | 1.04 (0.98, 1.10) | 1.02 (0.98, 1.06) |
| Female sex | 0.26 (0.10, 0.70)** | 0.85 (0.55, 1.30) | 2.17 (1.40, 3.36)** | 1.21 (0.67, 2.19) | 3.15 (2.20, 4.50)***^†^ |
| Education medium | 1.04 (0.42, 2.58) | 1.17 (0.78, 1.75) | 0.68 (0.46, 1.02) | 1.34 (0.74, 2.42) | 0.85 (0.57, 1.28) |
| Education high (ref.: low) | 1.08 (0.28, 4.19) | 1.36 (0.75, 2.47) | 1.11 (0.66, 1.86) | 0.90 (0.37, 2.21) | 0.39 (0.21, 0.72)** |
| Income | 1.07 (0.91, 1.26) | 0.89 (0.79, 1.01) | 1.01 (0.93, 1.08) | 1.07 (0.94, 1.22) | 0.92 (0.86, 0.99)* |
| ***Social factors*** |  |  |  |  |  |
| Marital status (ref.: single) | 0.46 (0.19, 1.11) | 0.61 (0.42, 0.86)** | 0.60 (0.40, 0.89)* | 1.06 (0.61, 1.86) | 0.87 (0.60, 1.25) |
| Social network | 0.98 (0.69, 1.40) | 0.94 (0.76, 1.15) | 0.91, 0.75, 1.11) | 1.29 (0.99, 1.68) | 0.78 (0.66, 0.92)** |
| ***Health factors*** |  |  |  |  |  |
| Cognitive functioning | 0.99 (0.87, 1.13) | 1.01 (0.95, 1.08) | 0.98 (0.91, 1.04) | 1.08 (0.99, 1.18) | 1.02 (0.96, 1.08) |
| Self-rated health | 0.99 (0.97, 1.02) | 1.00 (0.99, 1.01) | 1.01 (1.00, 1.02)* | 0.99 (0.98, 1.01) | 1.00 (0.99, 1.01) |
| ***Psychological factors*** |  |  |  |  |  |
| Motivation | 1.95 (1.01, 3.75)* | 1.18 (0.96, 1.46) | 1.12 (0.89, 1.42) | 1.29 (0.95, 1.76) | 1.23 (1.01, 1.49)* |
| Diet-specific self-efficacy | 1.24 (0.82, 1.87) | 0.98 (0.80, 1.21) | 1.10 (0.88, 1.40) | 1.08 (0.78, 1.49) | 1.00 (0.83, 1.21) |
|  |  |  |  |  |  |
| CI: confidence interval; OR: odds ratio. Unadjusted p-values: **p* < 0.05, ***p* < 0.01, ****p* < 0.001. Bonferroni-adjusted p-value: ^†^*p* < 0.005. | | | | | |

| **Supplemental Table 5.** Quantitative bias analysis for linear regression of factors associated with a healthy diet (total score). | | | | | | |
| --- | --- | --- | --- | --- | --- | --- |
|  | b | SE | 95% CI | | *p* |  |
|  |  |  | Lower | Upper |  |  |
| ***Sociodemographic factors*** |  |  |  |  |  |  |
| Age | 0.02 | 0.02 | -0.02 | 0.05 | 0.382 |  |
| Female sex | 0.41 | 0.18 | 0.04 | 0.77 | 0.029 | * |
| Education level medium | 0.09 | 0.18 | -0.26 | 0.44 | 0.618 |  |
| Education level high (ref.: low) | 0.23 | 0.28 | -0.33 | 0.78 | 0.421 |  |
| Income | -0.03 | 0.05 | -0.13 | 0.07 | 0.601 |  |
| ***Social factors*** |  |  |  |  |  |  |
| Married/cohabitating   (ref.: single) | -0.23 | 0.15 | -0.54 | 0.07 | 0.136 |  |
| Social network | 0.00 | 0.09 | -0.17 | 0.17 | 0.990 |  |
| ***Health factors*** |  |  |  |  |  |  |
| Cognitive functioning | 0.06 | 0.03 | 0.00 | 0.11 | 0.051 |  |
| Self-rated health | 0.00 | 0.00 | -0.01 | 0.01 | 0.792 |  |
| ***Psychological factors*** |  |  |  |  |  |  |
| Motivation | 0.25 | 0.09 | 0.07 | 0.44 | 0.009 | ** |
| Diet-specific self-efficacy | 0.25 | 0.12 | 0.02 | 0.48 | 0.031 | * |
| Results based on imputed data, applying a Delta-adjustment (δ = 0.2), assuming a 20% deviation of imputed values from the missing at random (MAR)-assumption. CI: confidence interval; unadjusted p-values: **p* < 0.05, ***p* < 0.01 | | | | | | |

| **Supplemental Table 6** Results of the linear regression of factors associated with a healthy diet (total score), pattern-mixture-model | | | | | | |  |
| --- | --- | --- | --- | --- | --- | --- | --- |
|  | b | SE | 95% CI | | *p* |  | |
|  |  |  | Lower | Upper |  |  | |
| ***Sociodemographic factors*** |  |  |  |  |  |  | |
| Age | 0.01 | 0.01 | -0.01 | 0.04 | 0.279 |  | |
| Female sex | 0.69 | 0.14 | 0.38 | 0.92 | <0.001 | *** | |
| Education level medium | 0.01 | 0.18 | -0.36 | 0.37 | 0.974 |  | |
| Education level high (ref.: low) | -0.01 | 0.21 | -0.42 | 0.41 | 0.977 |  | |
| Income | -0.05 | 0.03 | -0.11 | 0.02 | 0.140 |  | |
| ***Social factors*** |  |  |  |  |  |  | |
| Married/cohabitating   (ref.: single) | -0.15 | 0.11 | -0.38 | 0.08 | 0.193 |  | |
| Social network | 0.06 | 0.06 | -0.06 | 0.17 | 0.339 |  | |
| ***Health factors*** |  |  |  |  |  |  | |
| Cognitive functioning | 0.01 | 0.02 | -0.03 | 0.05 | 0.580 |  | |
| Self-rated health | 0.00 | 0.00 | 0.00 | 0.01 | 0.474 |  | |
| ***Psychological factors*** |  |  |  |  |  |  | |
| Motivation | 0.22 | 0.06 | 0.09 | 0.35 | 0.001 | ** | |
| Diet-specific self-efficacy | 0.33 | 0.07 | 0.20 | 0.46 | <0.001 | *** | |
| ***Missing values*** |  |  |  |  |  |  | |
| Missing value (ref.: no missing values) | -0.30 | 1.56 | -3.38 | 2.80 | 0.852 |  | |
| … Missing values*age | 0.00 | 0.02 | -0.04 | 0.04 | 0.936 |  | |
| Missing values*female sex | -0.02 | 0.22 | -0.45 | 0.41 | 0.927 |  | |
| Missing values*education level medium | 0.13 | 0.28 | -0.42 | 0.68 | 0.639 |  | |
| Missing values*education level high | 0.43 | 0.31 | -0.15 | 1.05 | 0.177 |  | |

SE: standard error; CI: confidence interval; ***p* < 0.01, ****p* < 0.001; “missing value” = 1 if values were missing on any of the components of the healthy diet-score (ref.: missing value = 0 if no values were missing for components of the healthy diet score).

| **Supplemental Table 7** Results of the linear regression of factors associated with a healthy diet (total score, modified) | | | | | | | |
| --- | --- | --- | --- | --- | --- | --- | --- |
|  | b | SE | 95% CI | | *p* |  |  |
|  |  |  | Lower | Upper |  |  |  |
| ***Sociodemographic factors*** |  |  |  |  |  |  |  |
| Age | 0.01 | 0.01 | -0.01 | 0.04 | 0.245 |  |  |
| Female sex | 0.66 | 0.12 | 0.43 | 0.90 | <0.001 | *** |  |
| Education level medium | 0.06 | 0.13 | -0.21 | 0.33 | 0.657 |  |  |
| Education level high (ref.: low) | 0.11 | 0.18 | -0.24 | 0.47 | 0.523 |  |  |
| Income | -0.05 | 0.03 | -0.11 | 0.02 | 0.146 |  |  |
| ***Social factors*** |  |  |  |  |  |  |  |
| Married/cohabitating   (ref.: single) | -0.16 | 0.11 | -0.39 | 0.06 | 0.157 |  |  |
| Social network | 0.07 | 0.06 | -0.05 | 0.18 | 0.251 |  |  |
| ***Health factors*** |  |  |  |  |  |  |  |
| Cognitive functioning | 0.02 | 0.02 | -0.03 | 0.06 | 0.455 |  |  |
| Self-rated health | 0.00 | 0.00 | 0.00 | 0.01 | 0.505 |  |  |
| ***Psychological factors*** |  |  |  |  |  |  |  |
| Motivation | 0.22 | 0.06 | 0.10 | 0.34 | <0.001 | *** |  |
| Diet-specific self-efficacy | 0.34 | 0.06 | 0.21 | 0.46 | <0.001 | *** |  |

SE: standard error; CI: confidence interval; ***p* < 0.01, ****p* < 0.001; Assessment of dairy products excluding consumption of cheese and cream cheese
